# Learnability of the LAHSHAL Classification for Oral Clefts: Results of an International Webinar

**DOI:** 10.1097/SCS.0000000000011355

**Published:** 2025-04-11

**Authors:** Ruben Pieter Houkes, Greet Hens, Christer Kubon, Arja Heliövaara, Kata Dávidovics, Ilze Akota, Sonja Lux, Jennifer Ruiz, Montserrat Munill, Marta Redondo Alamillos, Albert Malet Contreras, Corstiaan Breugem

**Affiliations:** * Amsterdam UMC, University of Amsterdam, Amsterdam, The Netherlands; † Department of Plastic Surgery, Emma Children's Hospital, Amsterdam, The Netherlands; ‡ Otorhinolaryngology—Head and Neck Surgery, University Hospitals KU Leuven, Leuven, Belgium; § Department of Plastic Surgery, Haukeland University Hospital, Bergen, Norway; ‖ Cleft Palate and Craniofacial Center, Helsinki University Hospital, Helsinki, Finland; ¶ Division of Pediatric Surgery, Traumatology, Urology and Pediatric Otolaryngology; Department of Pediatrics, University of Pécs, Pécs, Hungary; # Riga Stradins University, Institute of Stomatology, Department of Oral and Maxillofacial Surgery and Oral Medicine, Riga, Latvia; ** National Centre for Cleft Lip Palate and Craniofacial Anomalies, University Clinic of Oral and Maxillofacial Surgery, Paracelsus Private Medical University, General Hospital, Salzburg, Austria; †† Pediatric Maxillofacial Surgery Unit, Hospital Universitary Vall d’Hebron, Barcelona; ‡‡ Oral and Maxillofacial Surgery Service, University Hospital 12 de Octubre, Madrid; §§ Maxillofacial Surgery Unit, Pediatric Surgery Service, Hospital Sant Joan de Déu, Barcelona, Spain

**Keywords:** Classification, ERN CRANIO, LAHSHAL system, orofacial clefts, webinar

## Abstract

**Introduction:**

Orofacial clefts are common congenital anomalies with significant physical, emotional, and social challenges. Classification systems like LAHSHAL aim to standardize cleft descriptions, enhancing clinical communication and research. Despite its adoption by the European Reference Network on Craniofacial Anomalies (ERN Cranio), the system’s familiarity and learnability among health care providers remain uncertain.

**Methods:**

This study assessed the learnability of LAHSHAL through pre-webinar and post-webinar surveys with 29 cleft care specialists from 11 countries. Participants classified 10 cleft cases using LAHSHAL before and after an educational webinar. Inter-rater agreement was evaluated using Fleiss Kappa.

**Results:**

Pre-webinar inter-rater agreement was very low (κ=−0.015), reflecting inconsistent system use and limited prior experience, as 60% of participants had never used it. Post-webinar agreement improved significantly (κ=0.217; *P*<0.001), and all participants applied LAHSHAL classifications. However, variability persisted, particularly for complex clefts like those involving Simonart's bands.

**Discussion:**

The webinar improved familiarity and consistency in LAHSHAL usage, emphasizing the value of educational interventions. Nonetheless, post-webinar variability highlights the need for further training, especially for atypical cleft presentations. Although LAHSHAL shows potential for advancing cleft classification and research, improved guidelines and integration with systems like ICD-10 are required.

**Conclusion:**

The LAHSHAL system is a promising classification tool for clefts, but variability in its application indicates the need for ongoing training and refinement to ensure accurate, widespread use.

Orofacial clefts are among the most common congenital anomalies worldwide. They arise when the growing facial complex fails to fuse or differentiate properly during early fetal development.^1,2^ The prevalence of orofacial clefting worldwide is estimated to be between 3.4 to 22.9 per 10,000 live births, depending on geographic region, ethnicity, and socioeconomic status.^3–5^ In the case of non-syndromic clefts, which occur more often than syndromic clefts, these anomalies are thought to be of multifactorial etiology, with contributions from both genetic and environmental factors.^1,6^

Orofacial clefts may have significant consequences throughout a patient’s lifetime that may potentially impact quality of life. Depending on the cleft type, children born with orofacial clefts may encounter various difficulties beyond their initial surgical repair, such as speech, hearing, dentofacial development, and psychosocial problems. Long-term and multidisciplinary follow-up and treatment are therefore required.^7–12^

Orofacial clefts are heterogeneous in presentation and in subsequent impact on the patient. A proper description and classification of the cleft type and severity is thus important to be able to study the long-term consequences of the condition on the physical, emotional, and social development of the patient, and the outcomes of treatment.

Classification of oral clefts ensures consistent communication among healthcare providers and researchers. The ideal system for the classification of oral clefts would be one that is straightforward, precise and with a high intra-rater and inter-rater reliability. Currently, such a system does not exist.

Over the past 100 years, numerous classification systems for clefts have been proposed, based on anatomy and embryology, each offering a variety of ways to describe the location and extent of the cleft.^2,13–18^ These systems were developed to improve clinical communication and treatment planning; however, the numerous classification systems available have spawned a myriad of problems. This has resulted in the inability of cleft specialists to communicate efficiently between cleft teams and hospitals because of a lack of a widely accepted system. Furthermore, it hinders the compilation of big data in a standardized form for research and epidemiological studies. The lack of consistency also makes comparison of clinical outcomes through institutions and countries difficult, thus limiting global improvements in cleft services.

One of these classification systems is the LAHSHAL system.^17^ LAHSHAL is an acronym that represents the sites affected by cleft: lip (L), alveolus (A), hard palate (H), and soft palate (S). The acronym should be interpreted from the patient’s perspective, where the first part represents the right side of the patient, and the last part the left side. Complete clefts are described with a capital letter, and incomplete clefts with a lowercase one. Furthermore, an asterisk (*) indicates minimal clefting, whereas a dot (•/.) signifies that the anatomic feature is unaffected. In addition, a plus sign (+) can be noted in the L column when a skin band (e.g., a Simonart’s band) is present. For example, a complete bilateral cleft lip with an incomplete bilateral cleft alveolus and incomplete cleft of the soft palate would be classified through the LAHSHAL method as “La•s•aL”. Recently, the LAHSHAL system was adopted as an item in the Level 2 data set by the European Reference Network on Craniofacial Anomalies, ERN Cranio, and implemented for the classification of orofacial clefts in local registries by its member hospitals. The European Reference Networks (ERNs) are virtual networks designed to connect healthcare providers across Europe, enabling them to collaborate and share expertise to care for patients with rare or complex conditions that need highly specialized treatment. ERN Cranio focuses specifically on rare craniofacial anomalies, cleft lip and palate, and ear anomalies, working to advance research, diagnosis, and patient care in these areas as shown on their website: https://www.ern-cranio.eu/. The aim of the implementation of the LAHSHAL system is a higher uniformity of cleft classification and cleft registration across Europe and an improvement in the quality and consistency of the clinical data obtained for both patient care and research purposes. An important advantage of LAHSHAL is that it allows subphenotyping of clefts. Subphenotyping is considered important in measuring the outcome of cleft care.^19,20^

Although ERN Cranio adopted the LAHSHAL system, the feasibility to implement this classification system in daily practice on a European level has not been tested so far. So, it remains uncertain how familiar cleft caregivers in ERN-Cranio member hospitals are with this system, whether it is easily learnable and how reliable the generated data will be. The present study investigates the learnability of the LAHSHAL system by conducting an online survey among cleft caregivers. Hopefully, the level of familiarity of the participants with the LAHSHAL system pre-webinar and the adaptability of the system after an educational webinar will give insights into the possible obstacles of the implementation and reveal areas that may smoothen these obstacles through special training and education.

## MATERIALS AND METHODS

A webinar was conducted within the ERN Cranio group, for which 2 participants from each of the 30 ERN Cranio member hospitals or affiliated centers from 19 European countries were invited to participate. In total, 29 participants from 11 different countries attended the “The LAHSHAL system: Current state of affairs and its learnability, results of a European webinar among cleft specialists” webinar hosted in Amsterdam, the Netherlands on the June 5, 2024. In a cross-sectional setting this study consisted of 2 surveys, a pre-webinar and a post-webinar survey. In each survey, 10 pictures of cleft patients were presented to the participants (Supplemental Data Content 1, http://links.lww.com/SCS/H672). Each participant was asked to classify the presented case according to the LAHSHAL system by noting the LAHSHAL classification in a free-text box. Participants were asked to fill in the pre-webinar survey directly before the webinar. After the webinar, the participants were once again asked to complete the post-webinar survey using the same ten pictures.

We examined the consensus among participants regarding the LAHSHAL classification pre- and post-webinar. Only fully completed surveys were included into our analysis. Surveys were created and sent through Castor EDC. Inter-rater agreement was calculated with Fleiss Multirater Kappa scores by using SPSS (Statistical Program for Social Sciences, version 29; SPSS Inc.). The local Ethical Committee of the University of Amsterdam granted ethical approval for this study (2023.0507), and participants gave consent for processing all data pseudo-anonymously for scientific purposes. The reporting was in adherence with the STROBE checklist.

## RESULTS

In total 15 of the 29 participants (response rate of 51.7%) fully completed the questionnaire pre-webinar, as did 17 post-webinar (response rate of 58.6%). Most participants were from Spain (n=4; 26.7%), Austria (n=3, 20.0%) or the Netherlands (n=2, 13.3%). Concerning the specialty of the participants, maxillo-facial surgeons were the largest group (n=7, 46.7%) followed by plastic surgeons (n=4; 26.7%). When asked what the preferred timing of classification is in the participant’s cleft care team, most responded immediately after birth during the first visit to the cleft team (n=12; 80.0%). Two respondents noted that their team classified during the preoperative work-up of the primary surgery (13.3%) and 1 said that their team classified patients immediately after birth, but re-evaluates the given classification during the preoperative work-up for the primary surgery (6.7%). A majority of the participants said that one specific health care worker or specialist classifies new cleft patients in their cleft care team (60.0%), followed by 2 participants who mentioned that the cleft caregiver who first suspected the cleft anomaly classifies the patient for their team (13.3%) and 2 participants said that classification of new cleft patients was done through a multidisciplinary meeting in their hospital (13.3%). The specific specialist who most often classified cleft patients according to the participants was the maxillo-facial surgeon (n=4; 26.7%). When asked what classification system is used by their cleft care team, the ICD-10 system was mentioned by a majority (n=6; 40.0%). Other classification systems mentioned were the Veau classification (n=2; 13.3%) and the ACPA classification^15^ (n=2; 13.3%). Most respondents answered that they had not previously used the LAHSHAL classification system (n=9; 60.0%) versus a minority who did (n=6; 40.0%).

Agreement between the participants was pre-webinar very low (κ=−0.015; 95% CI: −0.035 to 0.006; *P*=0.163) but improved after the webinar (κ=0.217; 95% CI: 0.186–0.249; *P*<0.001). For example, pre-webinar 10 different responses were given when classifying the first case of the questionnaire concerning a patient with a right-sided complete cleft lip, alveolus, hard and soft palate, ranging from numerical scores to some sort of LAHSHAL classification, whereas post-webinar all the respondents classified the case using the LAHSHAL system, albeit with 6 different variations. This also occurred in the second case, concerning a patient with a bilateral complete cleft lip, alveolus and complete cleft of the hard and soft palate where respondents gave 6 different classifications consisting of LAHSHAL variations, anatomic descriptions and numerical scores. Post-webinar the respondents classified the case through 4 different LAHSHAL variations. In the ninth case, there was even perfect agreement between the respondents post-webinar whereas pre-webinar 9 different classifications were noted. The precise classifications noted by the various respondents both pre-webinar and post-webinar is shown in Supplemental Data Content 2, http://links.lww.com/SCS/H673 and Supplemental Data Content 3, http://links.lww.com/SCS/H674.

## DISCUSSION

The findings of this study underpin the difficulties and enhancements associated with the application of the LAHSHAL classification system for orofacial clefts, as demonstrated by pre-responses and post-responses against the cleft care provider educational webinar. First, low inter-rater agreement found pre-webinar (κ=−0.015) points to the variable use of cleft classification systems across institutions and countries. This inconsistency indicates that even though the LAHSHAL system exists and was adopted by ERN Cranio, the knowledge base of this classification tool among cleft care specialists is very low, as 60% of respondents have never used it before. This gap likely contributed to the inconsistency that was seen in the pre-webinar responses regarding classification, where often answers did not adhere to the LAHSHAL classification method. This also indicates that a substantial number of participants were not familiar with the LAHSHAL classification system.

The improved agreement after the webinar κ=0.217, *P*<0.001 would suggest that educational events such as the webinar may improve the cleft care providers’ familiarity with and correct use of the LAHSHAL system. It is clear that there was a reduced number of classification responses per case post-webinar. This shows that more extensive or intensive educational effort is needed to become fully acquainted with the LAHSHAL system, particularly with this wider diffusion of the LAHSHAL system within European countries. But even with this improvement, there remains considerable variability in the classifications even after the education intervention. Because of this rather limited improvement post-webinar, it remains questionable if the inter-rater agreement of the LAHSHAL system will be able to ever become very good, even after substantial educational efforts.

When comparing the results of this study to previous literature, remarkably there is mentioning of the implementation of the LAHSHAL classification system, but research on its learnability and inter-rater agreement analysis is scarce. For example, Houkes et al^21^ showed a significant implementation of the LAHSHAL system across international cleft care communities, especially in the United Kingdom. Another mention of the implementation of the LAHSHAL system has been done by Alyahhan et al^22^ In the Al-Ameri hospital in Kuwait, orofacial clefts are recorded with the use of the LAHSHAL system. Alghamdi et al^23^ describes the usage of the LAHSHAL system in 29 different government hospitals in Saudi Arabia. Some research on the learnability of LAHSHAL exists though. Wang and colleagues compared the learnability of both the LAHSHAL system and the RPL system among medical students. The RPL system is a 3-digit numerical framework used to specify the location of the cleft and the number of affected anatomic components, devised to be easily recorded for digital storage and analysis. Tests were administered immediately after a presentation of the 2 systems and after 2 weeks to assess the retention of the 2 systems. At both moments in time, the RPL system had a higher inter-rater agreement than the LAHSHAL system. A major limitation of the RPL system in comparison to the LAHSHAL system though, is its inability to take severity of the orofacial cleft into account.^24,25^

Complex cleft manifestations contributed to greater variability in classification among participants, even after the webinar intervention. The additional symbols used to classify minimal clefting (*), normal anatomy (•/.) or a Simonart’s band (+ noted in the L column), can be confusing and potentially contribute to the variability in the results. In our study, case 7, which featured a patient with a Simonart’s band, exemplified this difficulty. Despite the post-webinar improvement in consistency across most cases, classifications of case 7 continued to show significant variation (11 different classifications given by respondents post-webinar versus 12 pre-webinar). This persistent inconsistency highlights the challenge that atypical anatomic features pose to standardized classification systems like LAHSHAL. It suggests that certain cleft variations require additional guidelines or focused training to achieve a higher level of inter-rater agreement. However, it is clear that more training is mandatory before health care professionals can accurately use this classification system to compare outcomes.

The LAHSHAL system can represent a major move forward in the subphenotyping of the orofacial clefts, offering more uniformity and detail than traditional broad classification schemes. Application of this elaborate approach allows one to delineate small associations of specific cleft types with genetic or environmental factors. The LAHSHAL system thus allows a fine-grained look at cleft variation that can inform both genetic mutation studies and prenatal risk factor surveys, offering new knowledge concerning OFC etiology and supporting personalization of prevention and treatment.^20^ Moreover, LAHSHAL offers the advantage of compatibility with the ICD-10 system, whereby clefts have traditionally been represented by broad categories. Site-specific classification within LAHSHAL details specific anatomic features that are important for consistency in data, accuracy, and interoperability for clinical and research purposes. It will indeed be very useful to link LAHSHAL codes with ICD-10 to preserve such a high level of information in medical records, which would further enhance data exchange, clinical follow-up, and epidemiological research at an international level.

Several limitations occurred in this study. One of the main factors is the very low response rate. Although 60 cleft care specialists in a total of 30 centers were invited, only 29 participants took part, resulting in a response rate of 41.4%. This limited attendance reduces the generalizability of our findings because the smaller sample may not fully represent the range of experiences and familiarity with the LAHSHAL system across ERN cleft care centers. This poor response rate also indicates possible selection bias, with responders perhaps systematically different from nonresponders, and thus results may be biased toward those currently most interested in or using cleft classification systems. To tackle this problem centers were requested that 2 representatives attended this webinar. Nevertheless, from most centers only 1 representative was present. Future work will be strengthened by more diverse participation to enhance the generalizability and strength of results for the greater community involved in cleft care. It is positive that most centers indicated to have designated people to register the cleft and that most registration take place at a specific comparable time. Moreover, it will be interesting to see if the adjunct of Artificial intelligence to identify pictures could result in a more automated system and a more accurate classification.

In conclusion, subphenotyping an orofacial cleft is important for measuring outcomes. Therefore, a detailed description of the cleft is key for any registration system. For this purpose, the LAHSHAL system together with the ICD 10 offers a nice way to describe common orofacial cleft in detail. The drawback of registering more detail is the increased complexity of the classification used. This study provides insights into the learnability and applicability of the LAHSHAL system. Larger-scale educational campaigns, possibly aided by digital tools, and training simulations will be important to increase the reliability of LAHSHAL data and implement this system on a European level. Dedicated registration people per cleft team, might be required to achieve 100% consistency. Although this educational webinar improved inter-rater agreement among cleft caregivers, further work will be needed to discover how reliable the LAHSHAL system can become for classification and registration purposes of orofacial clefts through educational efforts.

## Supplementary Material

SUPPLEMENTARY MATERIAL
