# Supplementary material for: Learnability of the LAHSHAL Classification for Oral Clefts: Results of an International Webinar
Source: J Craniofac Surg. 2025 Apr 11;36(8):3032–5. doi: 10.1097/SCS.0000000000011355 (PMC12537025; doi:10.1097/SCS.0000000000011355)
Supplement: SUPPLEMENTARY MATERIAL [file scs-36-03032-s001.pdf]

Supplemental Data Content 1

LAHSHAL system questionnaire

Before you proceed to the questionnaire, we kindly request your approval for storing, analyzing, and utilizing the collected data to develop the results into a manuscript that will be submitted for publication. Your consent in this matter is greatly appreciated.

a. I consent
b. I do not consent

1. Hospital name

2. City

3. Country

4. Function
    a. Plastic surgeon
    b. Maxillo-facial surgeon
    c. Pediatrician
    d. Orthodontist
    e. ENT surgeon
    f. Clinical geneticist
    g. Dentist
    h. Speech and language therapist
    i. Nurse Practitioner

5. When do you classify clefts?
    a. Immediately after birth
    b. During the pre-operative work-up of the primary surgery
    c. During the primary surgery
    d. Other

6. Who records the classification of orofacial clefts? (Check all that apply)
    a. The specialist who first suspected the possibility of a cleft anomaly and performed diagnostic tests accordingly
    b. A specific specialist (for example: Pediatrician, surgeon, geneticist etc.)
    c. Through a multidisciplinary meeting
    d. Other

7. In case you answered the last question with 'a specific specialist', which specialist records at your team/institution the classification of clefts? (In case you did not answer the last question with 'a specific specialist', you can skip this question)

8. Did you already use the LAHSHAL system in clinical practice or for registration purposes, or have you started using it after the ERN Cranio meeting in which the centres agreed to use LAHSHAL for classification and registration purposes?

9. In case you did not use the LAHSHAL system previously, which system did you use in clinical practice and for registration purposes?
   a. Veau (Veau, 1932)
   b. Kernahan Y-striped (Kernahan, 1971)
   c. ACPA (Harkins et al., 1962)
   d. CLAP notation (Allori et al., 2017)
   e. Fogh-Andersen (Fogh-Andersen, 1971)
   f. Jensen (Jensen et al., 1988)
   g. Luijsterburg (Luijsterburg et al., 2014)

Please classify the following cases with the LAHSHAL classification system. You will first see the image and description of the cleft, and in the following step you can fill in the classification following the LAHSHAL method. **If a certain anatomical region is not displayed or described in the image or description (lip/alveolus/palate), you can assume that the region is not affected by the cleft.**

10. Case 1 - Please classify this case of a patient with a *right-sided complete cleft lip, alveolus, hard and soft palate*, following the LAHSHAL classification system.

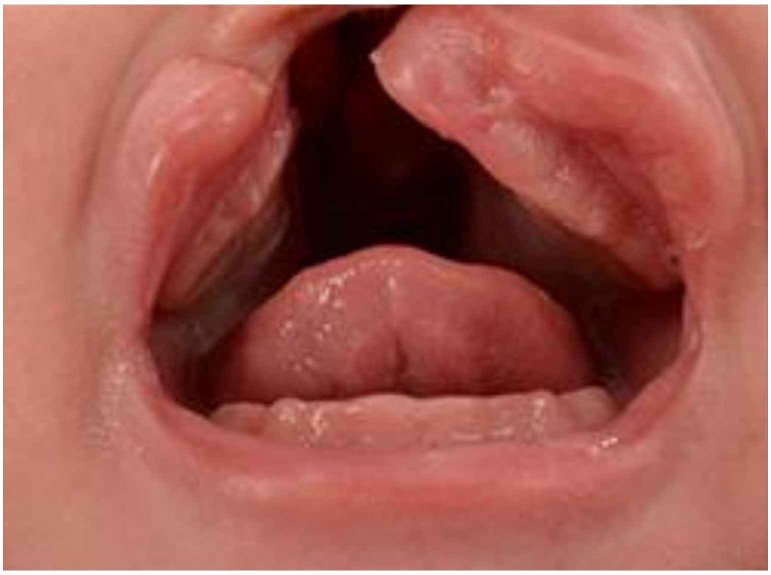

Correct LAHSHAL classification:

L	A	H	S	.	.	.

11. Case 2 - Please classify this case of a patient with a *bilateral complete cleft lip, alveolus and complete cleft of the hard and soft palate*, following the LAHSHAL classification system.

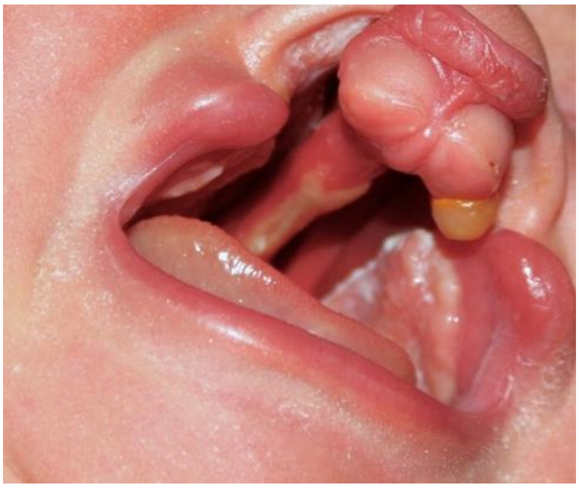

Correct LAHSHAL classification

L	A	H	S	H	A	L

12. Case 3 - Please classify this case of a patient with a *submucous cleft of the soft palate*, following the LAHSHAL classification system.

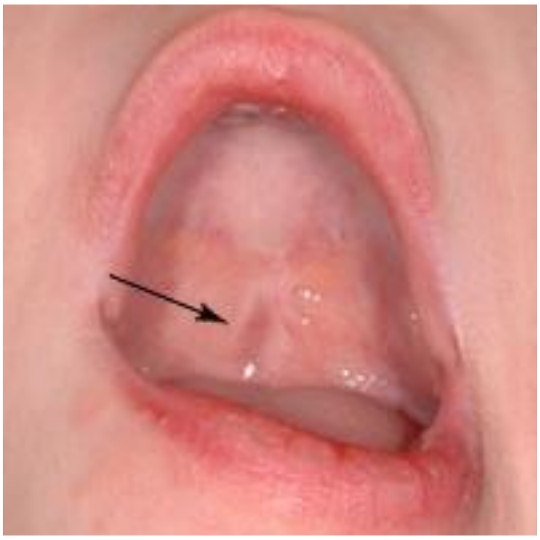

Correct LAHSHAL classification

.	.	.	*	.	.	.

13. Case 4 - Please classify this case of a patient with a *bilateral incomplete cleft lip*, following the LAHSHAL classification system.

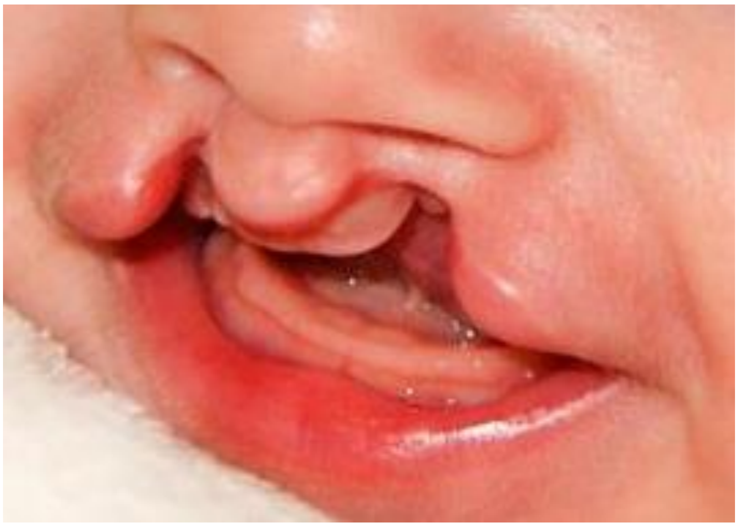

Correct LAHSHAL Classification

l	.	.	.	.	.	l

14. Case 5 - Please classify this case of a patient with a *right-sided incomplete cleft lip*, following the LAHSHAL classification system.

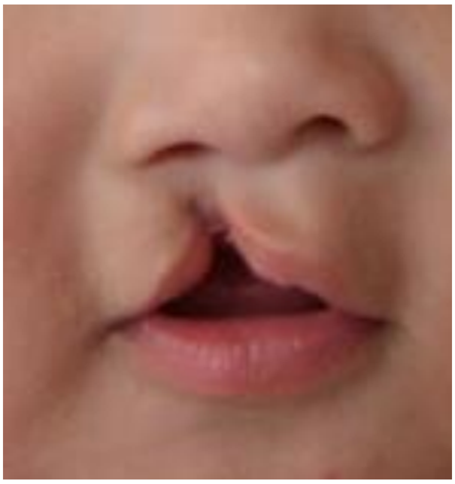

Correct LAHSHAL classification

l	.	.	.	.	.	.

15. Case 6 - Please classify this case of a patient with a *complete cleft of the soft palate and an incomplete cleft of the hard palate*, following the LAHSHAL classification system.

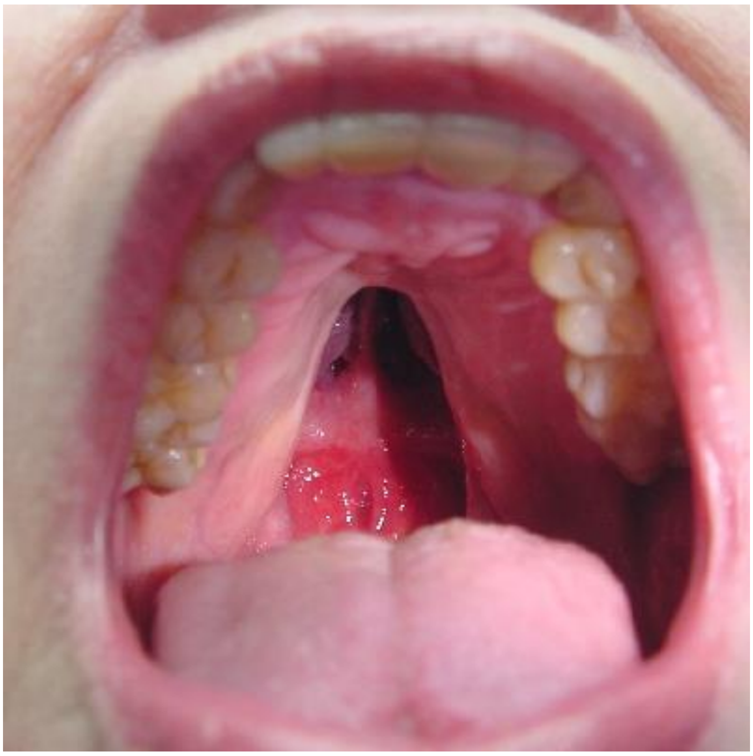

Correct LAHSHAL classification

.	.	h	S	h	.	.

16. Case 7 - Please classify this case of a patient with a *right-sided complete cleft lip, cleft aveolus, hard and soft palate with presence of a Simonart band*, following the LAHSHAL classification system.

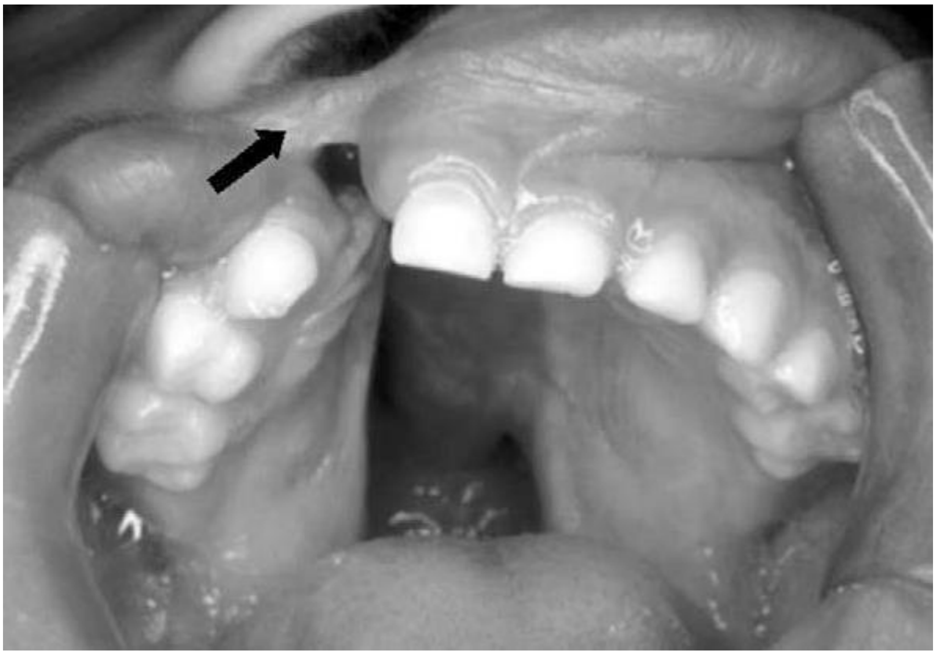

Correct LAHSHAL classification

L+	A	H	S	.	.	.

17. Case 8 - Please classify this case of a patient with a *complete cleft of the soft palate*, following the LAHSHAL classification system.

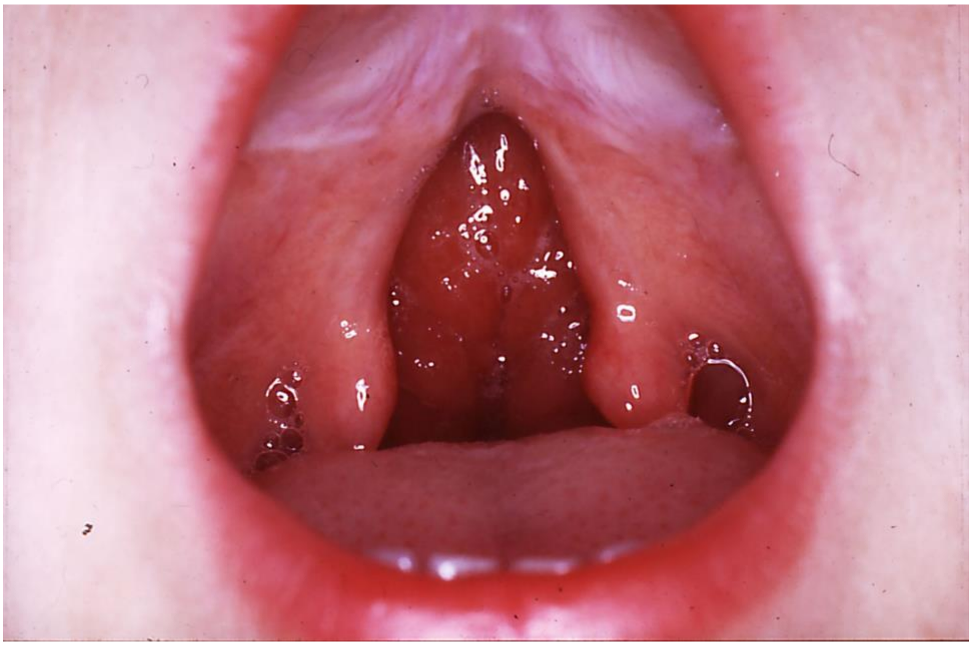

Correct LAHSHAL classification

.	.	.	S	.	.	.

18. Case 9 - Please classify this case of a patient with a *left-sided complete cleft lip and alveolus*, following the LAHSHAL classification system.

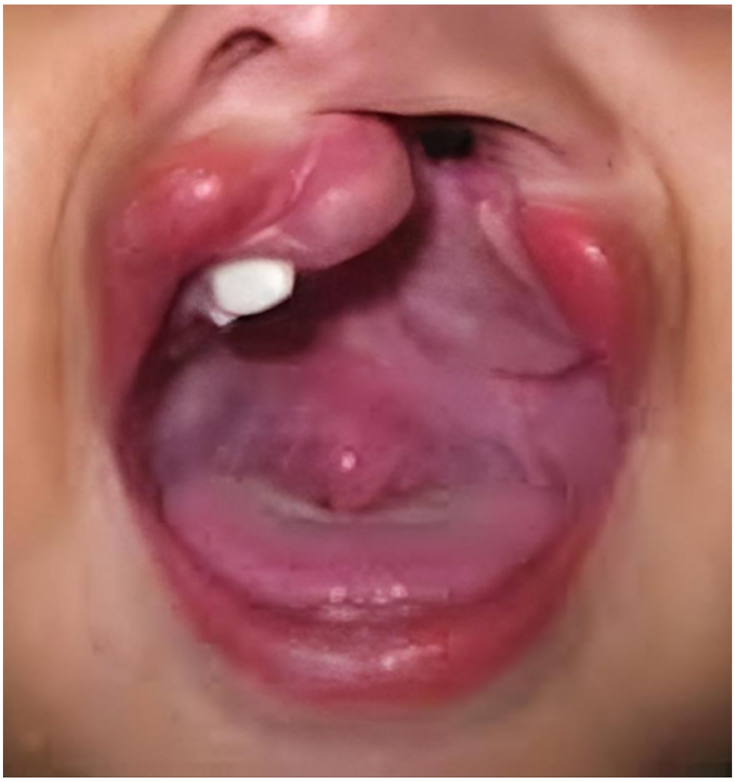

Correct LAHSHAL classification

.	.	.	.	.	A	L

19. Case 10 - Please classify this case of a patient with a *right-sided microform cleft lip*, following the LAHSHAL classification system.

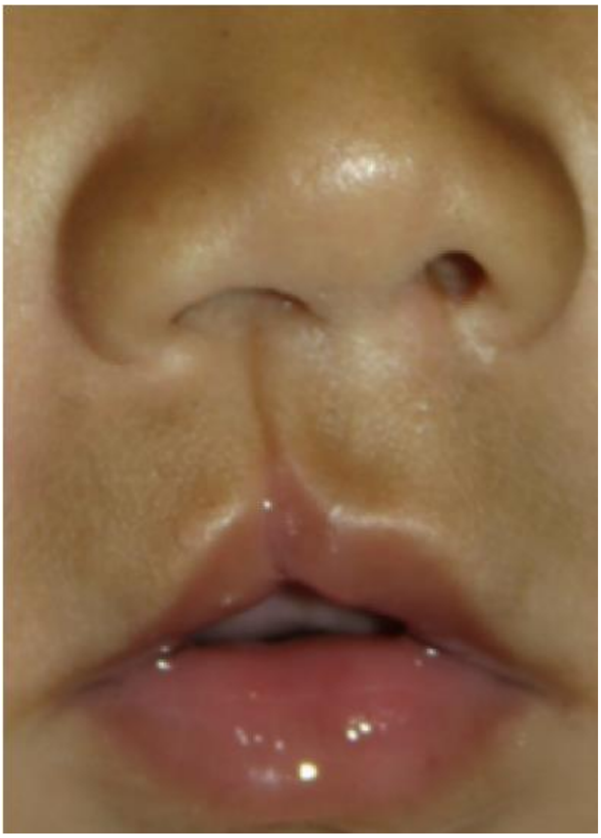

Correct LAHSHAL classification

*	.	.	.	.	.	.
