# Supplementary material for: Learnability of the LAHSHAL Classification for Oral Clefts: Results of an International Webinar
Source: J Craniofac Surg. 2025 Apr 11;36(8):3032–5. doi: 10.1097/SCS.0000000000011355 (PMC12537025; doi:10.1097/SCS.0000000000011355)
Supplement: SUPPLEMENTARY MATERIAL [file scs-36-03032-s002.pdf]

Supplemental Data Content 2

	Case 1 (LAHS…)	Case 2 (LAHSHAL)	Case 3 (…*…)	Case 4 (l…..l)	Case 5 (l……)	Case 6 (..hSh..)	Case 7 (L+AHS…)	Case 8 (…S…)	Case 9 (…..AL)	Case 10 (*……)
**1.**	LAHS… (n=6; 40.0%)	LAHSHAL (n=9; 60.0%)	…*… (n=2; 13.3%)	la…al (n=3; 20.0%)	l…... (n=4; 26.7%)	..HS… (n=3; 20.0%)	LAHS… (n=3; 20.0%)	S (n=4; 26.7%)	…..AL (n=5; 33.3%)	l…... (n=2; 13.3%)
**2.**	LAHSh.. (n=1; 6.7%)	123456789 (n=2; 13.3%)	S (n=2)	LAAL (n=2; 13.3%)	1 (n=2; 13.3%)	..HSH.. (n=2; 13.3%)	LASH… (n=2; 13.3%)	…S… (n=3; 20.0%)	AL (n=2; 13.3%)	*…... (n=2)
**3.**	LAHSH.. (n=1)	LAHS R LAHS L (n=1; 6.7%)	***s*** (n=2)	LAAL inkomplett (n=1; 6.7%)	L incomplete (n=1; 6.7%)	..hSh.. (n=2)	lAHSh.. (n=1; 6.7%)	89 (n=2; 13.3%)	456 (n=2)	1 (n=2)
**4.**	LAHS… R (n=1)	L14A25H36H78s9 (n=1)	9 (n=2)	l…..l (n=1)	La (n=1)	789 (n=2)	HHH (n=1)	HS (n=1; 6.7%)	LA (n=1; 6.7%)	L incomplete (n=1; 6.7%)
**5.**	L1A2H378S9 (n=1)	44444 (n=1)	S incomplete (n=1; 6.7%)	LA r LA l (n=1)	L r (n=1)	SH incomplete (n=1; 6.7%)	LAHS r (n=2)	S9 (n=1)	LA l (n=1)	l* (n=1)
**6.**	4440 (n=1)	bcCLAPv4 (n=1)	+s+ (n=1)	L14A25 (n=1)	LAH (n=1)	H78S9 (n=1)	L1A2 (n=1)	00200 (n=1)	L4A5 (n=1)	L r (n=1)
**7.**	ucCLAPv3 (n=1)		S9 (n=1)	30003 (n=1)	L1 (n=1)	00300 (n=1)	44400 (n=1)	***S*** (n=1)	0044 (n=1)	L1 (n=1)
**8.**	123789 (n=1)		00100 (n=1)	biCLA (n=1)	30000 (n=1)	**hSh** (n=1)	lAHS*** (n=1)	v2CP (n=1)	*****AL (n=1)	10000 (n=1)
**9.**	R123789 (n=1)		v1sm (n=1)	l*HSAl (n=1)	l****** (n=1)	v2CP (n=1)	ucCLAPv4 (n=1)	..HSH.. (n=1)	ucCLA (n=1)	l****** (n=1)
**10.**	LAHShal (n=1)		…s… (n=1)	123456 (n=1)	uiCLA (n=1)	…SH.. (n=1)	123789 (n=1)			umCL (n=1)
**11.**			? (n=1)	1245 (n=1)	L (n=1)		23789 (n=1)			L (n=1)
**12.**				l----al (n=1)			? (n=1)			? (n=1)

Pre-webinar results. Classifications noted ranked by frequency used per case. The correct LAHSHAL classification is shown in brackets in the box of the corresponding case.
