# Supplementary material for: Learnability of the LAHSHAL Classification for Oral Clefts: Results of an International Webinar
Source: J Craniofac Surg. 2025 Apr 11;36(8):3032–5. doi: 10.1097/SCS.0000000000011355 (PMC12537025; doi:10.1097/SCS.0000000000011355)
Supplement: SUPPLEMENTARY MATERIAL [file scs-36-03032-s003.pdf]

	Case 1 (LAHS…)	Case 2 (LAHSHAL)	Case 3 (…*…)	Case 4 (l…..l)	Case 5 (l……)	Case 6 (..hSh..)	Case 7 (L+AHS…)	Case 8 (…S…)	Case 9 (…..AL)	Case 10 (*……)
**1.**	LAHS… (n=13; 76.5%)	LAHSHAL (n=13; 76.5%)	…*… (n=9; 52.9%)	l…..l (n=7; 41.2%)	l…… (n=16; 94.1%)	..hSh.. (n=11; 64.7%)	'+AHS... (n=5; 29.4%)	…S… (n=13; 76.5%)	…..AL (n=17; 100.0%)	*...... (n=9; 52.9%)
**2.**	LAHSH.. (n=2; 11.8%)	LAHSAL (n=3; 17.6%)	…s… (n=5; 29.4%)	la…al (n=6; 35.3%)	l*a..... (n=1; 5.9%)	..HSH.. (n=3; 17.6%)	L+AHS… (n=3; 17.6%)	..hSh.. (n=3; 17.6%)		*l…… (n=3; 17.6%)
**3.**	LAHSh.. (n=1; 5.9%)	LAHSHSAL (n=1; 5.9%)	…s*… (n=1; 5.9%)	l*...al (n=1; 5.9%)		..HS.. (n=1; 5.9%)	'+LAHSH.. (n=1; 5.9%)	..HSH.. (n=1; 5.9%)		l+..... (n=2; 11.8%)
**4.**	LASH… (n=1)		…S… (n=1)	lA...Al (n=1)		..HsH.. (n=1)	*LAHS… (n=1)			l...... (n=2)
**5.**			... S*... (n=1)	l….al (n=1)		..lSl.. (n=1)	AL+.... (n=1)			L…..* (n=1; 5.9%)
**6.**				(n=1)			LAHS…+ (n=1)			
**7.**							l+AHSH.. (n=1)			
**8.**							+LAHSh.. (n=1)			
**9.**							LAHS...+ (n=1)			
**10.**							L+AHAH.. (n=1)			
**11.**							lAHS… (n=1)			

Post-webinar results. Classifications noted ranked by frequency used per case. The correct LAHSHAL classification is shown in brackets in the box of the corresponding case.
